# Spontaneous remission of angioimmunoblastic T-cell lymphoma in a child with ataxia–telangiectasia: a case report

**DOI:** 10.1186/s13256-023-04050-5

**Published:** 2023-08-04

**Authors:** Dima Abla, Abeer Al-Battashi, Khalil Albiroty, Khuloud Abu Qasida, Nasser Al-Rahbi, Salah Al-Awaidy

**Affiliations:** 1 Department of Pediatric Hematology and Oncology, The National Oncology Centre, Royal Hospital, Ministry of Health, Muscat, Oman; 2 Department of Pathology, Royal Hospital, Ministry of Health, Muscat, Oman; 3 Health Affairs, Ministry of Health, P. O. Box 393 PC 100, Muscat, Oman

**Keywords:** Angioimmunoblastic T cell lymphoma, Spontaneous regression, Pediatric patient, Case report, Oman

## Abstract

**Background:**

Angioimmunoblastic T-cell lymphoma is an uncommon subtype of peripheral T-cell lymphoma in children with fewer than 20 cases reported in literature.

**Case presentation:**

A 3-year-old Omani boy was diagnosed with ataxia–talengectasia presenting with fever and generalized lymphadenopathy. His biopsy revealed atypical lymphocytic infiltrate consistent with the diagnosis of angioimmunoblastic T-cell lymphoma. Within 3 weeks from the initial presentation and without any neoadjuvant therapy, he showed complete recovery of symptoms with absence of fever and regression of all previously affected lymph nodes. He has remained in remission ever since.

**Conclusion:**

This is the first report of spontaneous improvement of angioimmunoblastic T-cell lymphoma in a patient with ataxia–telangiectasia who was 3 years old at presentation. Owing to the paucity of similar cases, this report adds valuable diagnostic, therapeutic, and monitoring data.

## Background

Peripheral T-cell lymphomas (PTCLs) are extremely rare in the childhood age group. They represent less than 2% of all childhood non-Hodgkin’s lymphoma (NHL) [1] and are divided into 28 subtypes as per the 2016 World Health Organization classification system [2]. Although the outcome of pediatric patients with PTCL appears to be inferior to that of adult patients, the survival rates are still poor compared with other types of NHL [1]. The management of this type of lymphomas in children remains challenging, and no standard treatment strategy has been defined [3].

We present a case of AITL in a 3-year-old male child with ataxia–telangiectasia who improved spontaneously without any neoadjuvant chemotherapy.

## Case report

### Presentation and clinical characteristics

In February 2018, medical advice was sought for a 3-year-old Omani male, previously healthy patient, owing to unstable gait and recurrent upper respiratory infections. The diagnosis of ataxia–telangiectasia was confirmed at that time by identifying a novel homozygous mutation in the *ATM* gene. Family history was unremarkable, and parents were first-degree relatives. Other sibling are fine, except his younger sister was diagnosed last year (2022) with same condition.

He was started on treatment with monthly and lifelong intravenous immunoglobulin at a prophylactic dose of 0.4 milligrams per kilogram.

In May 2018 he presented with 1-week history of fever and cough. Upon presentation, he was febrile with average temperature of 38.7 ℃, heart rate of 110 beats per minute, and blood pressure within normal limits for his age and sex. On physical examination, he had submandibular, cervical, axillary, and inguinal enlarged lymph nodes (largest was 3 × 3 cm in the right axilla) with no detectable hepatosplenomegaly. Neurological examination revealed nystagmus and an unbalanced gait. Romberg sign was negative, and deep reflexes were normal.

### Investigations

An abdominal ultrasound was unremarkable for any intraabdominal lesions or organomegaly. Radiation in the form of X-ray or computed tomography was avoided due to his genetic background. The blood tests revealed severe neutropenia (neutrophil count 0.2 × 10^9^/L) with total white blood cells of 3.9 × 10^9^/L, hemoglobin of 9 g/L, and platelet count of 214 × 10^9^/L. His lactate dehydrogenase was 275 U/L, and Epstein–Barr virus (EBV) was undetectable by polymerase chain reaction (PCR). Other blood tests including liver function and renal function tests were all within normal ranges for his age. The patient was started on general supportive care with intravenous hydration, antipyretics (paracetamol 15 milligrams per kilogram intravenously), and intravenous ceftriaxone at a dose of 50 milligrams per kilogram twice a day for 5 days. Owing to the presence of lymphadenopathy, neutropenia, and high suspicion of malignancy in patients with primary immunodeficiency, the patient underwent a right axillary lymph node excisional biopsy.

### Pathology

**Fig. 1:**
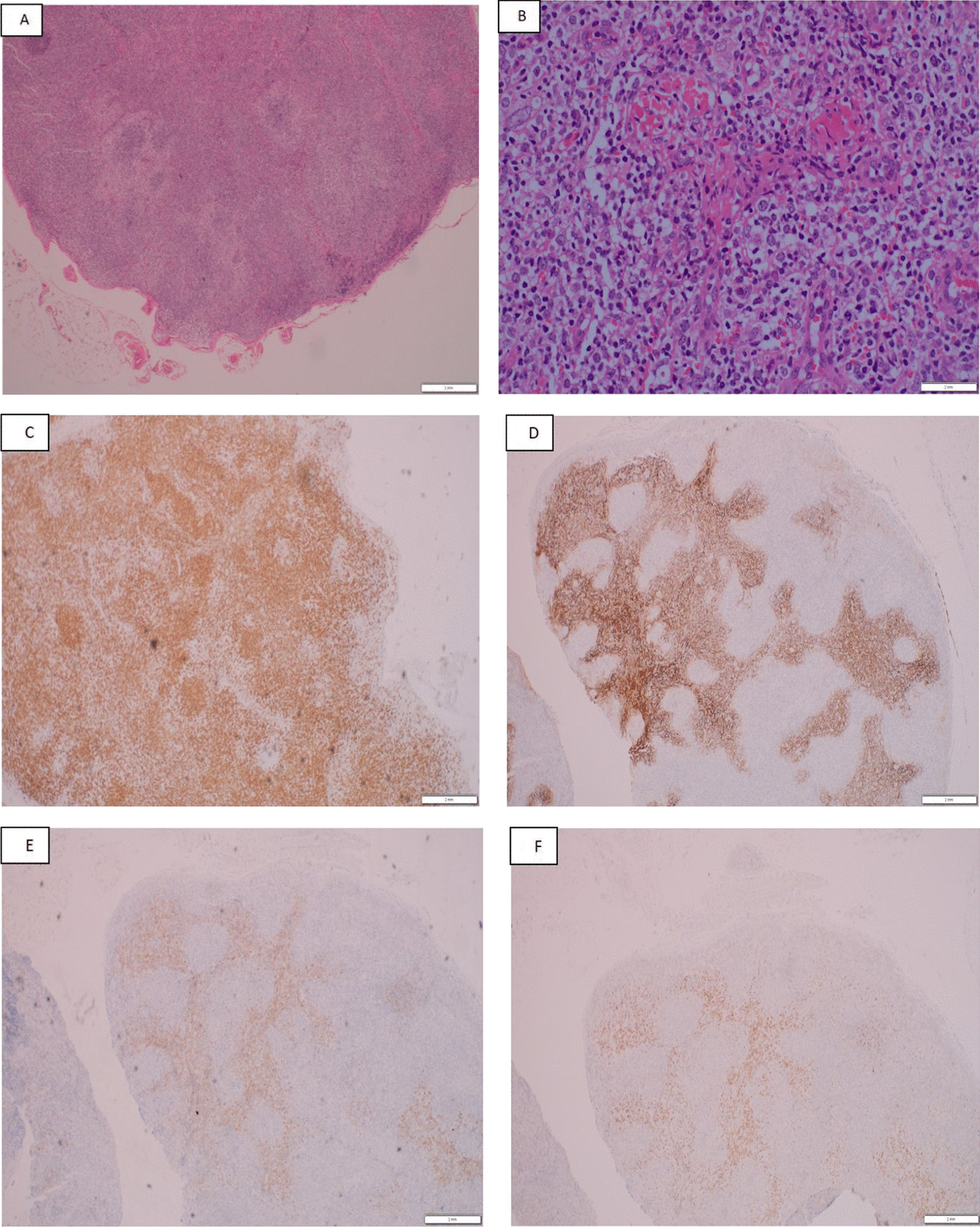
The lymph node biopsy findings and immunophenotypic features: effacement of architecture hematoxylin–eosin (H&E) stain, 4× (**A**). There are atypical lymphoid cells with high endothelial venules proliferation. H&E stain, 20×. **B** The neoplastic lymphocytes are positive for CD3, 10× (**C**). CD21 highlights the expanded follicular dendritic meshwork, 4×. **D** The neoplastic cells are positive for T follicular helper cell markers including CD10, BCL6 (**E**, **F**, respectively, 4×), and PD1 (not shown)

Microscopic analysis demonstrated infiltration with atypical lymphoid cells having small, medium to large nuclei and clear cytoplasm localized around high endothelial venules. Immunohistochemical stains showed positivity of the atypical lymphoid cells for CD3, CD4, CD10, BCL6, and PD1 (Fig. 1). The morphology and immunophenotype were both suggestive of AITL. The diagnosis was confirmed by molecular studies, which revealed detection of clonal T cell receptor gamma chain gene rearrangement as well as clonal B cell IgH/Kappa chain gene rearrangement. Bone marrow examination did not show any abnormal cellular infiltration.

## Follow‑up care and monitoring

The patient went into complete resolution of lymphoma symptoms with remittance of fever and resolution of the generalized lymphadenopathy within 3 weeks from the start of the acute condition. A decision was taken to manage the patient conservatively with close observation and follow‑up after extensive discussion with the parents and additional local departmental multidisciplinary team reviews. Since then, the patient has remained free of symptoms, apart from on-and-off EBV reactivation owing to his immunological background which was treated with rituximab (375 mg per square meter every week) for a total of four doses as per the clinical indications. He was cautiously followed up with physical examination, serial ultrasounds, and blood investigations including a full blood count, a blood film, and lactate dehydrogenase (LDH) levels. Currently this patient is 5 years from the AITCL diagnosis with no evidence of disease. During clinical follow-up, he started developing conjunctival talengectasia, which manifested around the age of 4 years as a natural course of the disease, but with no skin manifestation.

## Discussion

AT is a rare autosomal recessive genetic condition that is defined by the occurrence of cerebellar atrophy with progressive ataxia, and cutaneous telangiectasias [4]. In addition, patients are also characterized by unusual radiosensitivity and a high risk of development of malignancy (particularly lymphoid malignancy), immune deficiency, and recurrent sinopulmonary infections [4].

This is one of a few cases reported in literature about AITL in pediatric patients in general, and among AT patients in particular [1]. What adds to the peculiarity of this child’s condition is the spontaneous remission with no neoadjuvant therapy whatsoever. In fact, cytotoxic therapy could add a significant long-term toxicity and a more cancer risk in a child who is already predisposed due to his genetic frame.

PTCL is a very rare, aggressive type of NHL in children [1]. It originates from mature T cells in the thymus and has rearrangement of T-cell receptor genes. The most common subtype in children is peripheral T-cell lymphoma–not otherwise specified [5]. Other reported phenotypes can include hepatosplenic T-cell lymphoma, subcutaneous panniculitis-like T-cell lymphoma, extranodal NK/T-cell peripheral T-cell lymphoma, and AITL [5]. AITL, formerly known as angioimmunoblastic lymphadenopathy with dysproteinemia (AILD), was believed to be a benign immune response. It is currently recognized as a subtype of PTCL [6].

Very few cases of pediatric patients with AITL have been reported in literature [1]. The first case was reported in 1976 by Howarth and Bird. They described a 7-year-old male who presented initially with fever and generalized lymphadenopathy and then died due to disease progression 13 months after diagnosis [7]. In 1981, Fiorillo *et al*. reported the first case of AILD in childhood which underwent spontaneous improvement [8]. In 1989, Terlizzi *et al*. observed the same entity in a 14-month-old baby who had a fatal outcome [9]. Horneff *et al.* described neurological complications in a 13-year-old girl with AILD [10]. This is the first case report describing AITL in a pediatric patient in the context of AT that evolved toward spontaneous remission. Characteristics, treatment, and outcome of previously published patients are presented in Table 1.

**Table 1:** Characteristics, treatment, and outcome of pediatric patients with angioimmunoblastic lymphoma

Study	Number of patients	Age	Sex	Treatment	Outcome
Howarth and Bird [7]	1	7 years	Male	Prednisolone and vincristine	D
Fiorillo *et al*. [8]	1	8 years	Male	Supportive	L
de Terlizzi *et al.* [9]	1	14 months	Male	Prednisolone and vincristine	D
Horneff *et al.* [10]	1	13 years	Female	Prednisolone	L
Kobayashi *et al.* [11]	1	14 years	Male	JACLS NHL98T	L
Windsor *et al.* [5]	3	14 years	Female	Prednisolone	D
		7 years	Male	UKALL X	L
		3 years	Male	CHOP	D
Mellgren *et al.* [15]	4	Median 12.5 years	3 males 1 female	NR	3 L 1 D
Kraus *et al.* [20]	1	8 years	Male	Cyclophosphamide Etoposide Vincristine Prednisone	L
Our case	1	3 years	Male	Observation	L

*NR* not reported, *D* death, *L* alive

There is no consensus about the optimal treatment for pediatric PTCL owing to the absence of randomized clinical trials and the rarity of cases in pediatric patients [3]. Chemotherapy options include NHL or acute lymphoblastic leukemia (ALL) regimens [11]. The United Kingdom Children’s Cancer Study Group (UKCCSG) reported a superior outcome of ALL-like therapy compared with NHL therapy [4]. Adult studies suggest that CHOP-based first-line chemotherapy regimen consisting of cyclophosphamide, prednisolone, vincristine, and doxorubicin is superior and effective, but prognosis in adults remained dismal [12].

The association between other types of NHL and immune deficiency is well known. Many cases of AILD/AITL after immunosuppressive therapy have been reported in adults [13, 14]. In the retrospective EICNHL/i-BFM analysis of 143 children, preexisting conditions such as primary immune deficiency, immune suppressive therapy, and/or previous transplantation were found in 25% of the patients. It was also noticed that better outcome was observed in those patients [15]. Our patient was diagnosed with ataxia–telangiectasia months before the development of AITL.

Spontaneous regression of malignancies is a fascinating phenomenon. It is defined as the complete or partial disappearance of cancer without the use of any anti-neoplastic therapy [16]. It was first described by Sir William Osler in 1906 [17] and has been reported in patients with low-grade non-Hodgkin’s lymphoma [18]. The exact mechanism of this phenomenon remains unclear and most likely can be explained by undetermined immunological factors [19]. Among pediatric patients with PTCL, only 12 cases with spontaneous regression were reported in literature (Table 2).

**Table 2:** Characteristics and subtype of pediatric patients with PTCL and spontaneous regression

Study	Number of patients	Age	Sex	Subtype	Outcome
Fiorillo *et al.* [8]	1	8 years	Male	AILD	L
Windsor *et al.* [5]	1	9 years	Male	Angiocentric	L
Kobayashi *et al.* [11]	1	7 years	Male	PTCL-NOS	L
Maciejka-Kemblowska *et al.* [3]	1	16 years	Male	SPL	D
Mellgren *et al.* [15]	8	NR	NR	MF, PTCL NOS, SPL	L
Our case	1	3 years	Male	AITL	L

*NR* not reported, *AILD* angioimmunoblastic lymphadenopathy with dysproteinemia, *PTCL*-*NOS* peripheral T-cell lymphoma–not otherwise specified, *SPL* subcutaneous panniculitis-likeT-cell lymphoma, *MF* mycosis fungoides, *AITL* angioimmunoblastic T-cell lymphoma, *D* death, *L* alive

The outcome of our case can add confidence to treating pediatric oncologists when watchful waiting in the context of clinical improvement seems to be just right. Of course, this should not be the case for patients with PTCL who have active disease or signs of deterioration. The decisions must always be taken individually with caution based on clinical condition, comorbidities, and the course of the disease. Such decisions must always be made in a multidisciplinary approach within the treating teams.

## Conclusion

This is the first case report of spontaneous remission of angioimmunoblastic T-cell lymphoma in a 3-year-old patient with genetically confirmed ataxia–telangiectasia. This case study adds diagnostic, therapeutic, and monitoring knowledge in regard to this rare subtype of lymphoma. Owing to the paucity of cases of PTCL in children, international collaborative rials are indeed highly beneficial to establish treatment recommendations.

## Data Availability

Data sharing is not applicable to this article as no datasets were generated or analyzed during the current study.

## Abbreviations

AITL: Angioimmunoblastic T-cell lymphoma
PTCLs: Peripheral T-cell lymphomas
NHL: Non-Hodgkin’s lymphoma
EBV: Epstein–Barr virus
